# Cannabis Indica, a Valuable Remedy in Menorrhagia

**Published:** 1883-11

**Authors:** 


CANNABIS INDICA; A Valuable Remedy in Menorrhagia. Mr. J. BROWN, of Bacup, observes :

"Indian hemp has been vaunted as an anodyne and hypnotic, having the good qualities of opium without its evils. In dysmenorrhœa and insomnia it has not proved of much benefit. The drug has almost invariably produced some marked physiological effect even in small doses. Text books give the dose as ten minims and upwards, but five minims is the largest dose that should be given at first. If bought from a good house the drug is not inert and unreliable. A drug having such marked physiological action ought to have a specific use as a therapeutic agent. Indian hemp has such specific use in menorrhagia—there is no medicine which has given such good results ; for this reason it ought to take the first place as a remedy in menorrhagia, then bromide of potassium and other drugs. The *modus operandi* I can not explain unless it be that it diverts a large proportion of blood to the brain, and lessens the muscular force of the heart. A few doses are sufficient; the following is the prescription: ℞ tincturæ cannabis indicæ ♏xxx; pulveris tragac. co. ʒj; spiritus chlorof. ʒj; aquæ ad ℥ij. One, once every three hours. Four years ago I was called to see Mrs. W., aged 40, multipara. She had suffered from menorrhagia for several months. Her medical attendant had tried the ordinary remedies without success. Indian hemp was given as above. Its action was speedy and certain. Only one bottle was taken. She was afterwards treated for anæmia, due to loss of blood. Twelve months after this my patient sent for a bottle of the "green medicine." I learned afterwards that she had sent this medicine to a lady friend, who had been unsuccessfully treated by another medical man for several months for the same complaint. It proved equally successful. The failures are so few that I venture to call it a specific in menorrhagia. The drug deserves a trial. It may occasionally fail; this, however, is not to be wonderee at in a complaint due to so many different causes, and associated with anæmia and other cases of plethora."

